# Polyethylene Glycol 35 (PEG35) Modulates Exosomal Uptake and Function

**DOI:** 10.3390/polym12123044

**Published:** 2020-12-18

**Authors:** Ana Ferrero-Andrés, Daniel Closa, Joan Roselló-Catafau, Emma Folch-Puy

**Affiliations:** 1 Experimental Pathology Department, Institut d’Investigacions Biomèdiques de Barcelona-Consejo Superior de Investigaciones Científicas (IIBB-CSIC), 08036 Barcelona, Spain; ana.ferrero@iibb.csic.es (A.F.-A.); daniel.closa@iibb.csic.es (D.C.); joan.rosello@iibb.csic.es (J.R.-C.); 2 Institut d’Investigacions Biomèdiques August Pi i Sunyer (IDIBAPS), 08036 Barcelona, Spain

**Keywords:** polyethylene glycol, exosomes, pancreatitis, inflammation, macrophages, cytokines, NFκB

## Abstract

Polyethylene glycols (PEGs) are neutral polymers widely used in biomedical applications due to its hydrophilicity and biocompatibility. Exosomes are small vesicles secreted by nearly all cell types and play an important role in normal and pathological conditions. The purpose of this study was to evaluate the role of a 35-kDa molecular weight PEG (PEG35) on the modulation of exosome-mediated inflammation. Human macrophage-like cells THP-1, epithelial BICR-18, and CAPAN-2 cells were exposed to PEG35 prior to incubation with exosomes of different cellular origins. Exosome internalization was evaluated by confocal microscopy and flow cytometry. In another set of experiments, macrophages were treated with increasing concentrations of PEG35 prior to exposure with the appropriate stimuli: lipopolysaccharide, BICR-18-derived exosomes, or exosomes from acute pancreatitis-induced rats. Nuclear Factor Kappa B (NFκB) and Signal transducer and activator of transcription 3 (STAT3) activation and the expression levels of pro-inflammatory Interleukin 1β (IL1β) were determined. PEG35 administration significantly enhanced the internalization of exosomes in both macrophages and epithelial cells. Further, PEG35 ameliorated the inflammatory response induced by acute pancreatitis-derived exosomes by reducing the expression of IL1β and p65 nuclear translocation. Our results revealed that PEG35 promotes the cellular uptake of exosomes and modulates the pro-inflammatory effect of acute pancreatitis-derived vesicles through inhibition of NFκB, thus emphasizing the potential value of PEG35 as an anti-inflammatory agent for biomedical purposes.

## 1. Introduction

Polyethylene glycols (PEGs) are neutral polymers composed of repeated ethylene glycol units with different chain lengths [1]. PEGs are one of the best-investigated polymers with a large number of applications in different fields. The water-solubility and hydrophilic properties of PEGs combined with a low intrinsic toxicity make them ideally suited for biological applications. Indeed, their use is approved by the Food and Drugs Administration (FDA) and European Medicines Agency (EMA) in several fields such as the pharmaceutical, cosmetic, packing, food, and clinical fields [2].

Evidence from different experimental studies has suggested beneficial effects of PEGs in different pathological situations. PEGs have demonstrated membrane-protective effects in a variety of cells or organs against various insults. In this sense, PEGs reduce oxidative stress mainly through preservation of cell membrane integrity and protection against reactive oxygen species production during ischemia and reperfusion injury [3]. PEG ameliorated the early and late effects of radiation in mice intestine through the stabilization and preservation of lipid-raft signaling, leading to preservation of membrane integrity [4]. PEG has also been shown to reinforce epithelial barriers and to reduce inflammation of the colon in experimental colitis [5]. The intravenous administration of PEG is effective in minimizing myocardial ischemia-reperfusion injury and in preserving ventricular function through the inhibition of apoptotic signaling and upregulation of cell survival signaling [6]. Finally, the potential role of PEG in organ preservation has been demonstrated in experimental kidney [3], liver [7], and pancreatic [8] transplantation. The presence of PEG in the preservation solution ameliorated the deleterious consequences of ischemia-reperfusion by triggering protective cell signaling pathways.

However, there is much less information regarding the precise mechanisms involved in the described beneficial effects of PEGs. In particular, little is known about interactions between PEGs and the different biological processes mediated by extracellular vesicles and, more specifically, exosomes. Exosomes are the smallest subtype of extracellular vesicles secreted constitutively by fundamentally all cells in physiological conditions into the extracellular space [9]. They have received increasing attention since they were discovered to enclose functional proteins, lipids, DNA, mRNA, microRNA, and a large variety of other small noncoding RNA species [10,11]. Such exosomal cargo can be delivered into neighboring and distal cell subpopulations to either confer pathogenic or therapeutic effects through modulation of immune responses. Moreover, exosomes can be localized in many biological body fluids including blood, saliva, ascitic fluid, cerebrospinal fluid, and urine, thereby facilitating intercellular communication [12].

Over the past years, a number of studies have provided evidence of exosomes’ implication in physiological and pathological cellular events [13]. It has been reported that exosomes play relevant roles in processes as cancer, metastasis, neurological disorders, or inflammation. Previous studies of our group have determined the involvement of exosomes in lung damage associated with experimental acute pancreatitis (AP). Circulating exosomes from AP-induced rats reached the alveolar compartment and polarized macrophages to a pro-inflammatory phenotype [14]. Hence, exosomes are now recognized as promising targets in a large number of pathologies and any agent with capability to modulate their function can be considered a potential therapeutic tool.

Interestingly, we have recently reported an anti-inflammatory role of 35-kDa molecular weight PEG (PEG35) in experimental AP and associated lung injury [15]. Intravenous administration of PEG35 was able to reduce the severity of the inflammatory damage and to improve outcomes when administered following the initiation of AP-associated systemic effects. These effects may be attributed to the direct anti-inflammatory activity of PEG35, but a potential effect of this polymer on circulating exosomes could also be hypothesized.

In the present paper, we aimed to explore the interaction between exosomes and PEG35 in differentiated inflammatory cells in order to determine the potential role of PEG35 on the regulation of exosome uptake and function.

## 2. Materials and Methods 

### 2.1. Cell Culture 

The human pro-monocytic THP-1 cell line was purchased from Sigma-Aldrich (St. Louis, MO, USA). The cells were grown at 37 °C in suspension in RPMI 1640 medium GlutaMAX™ (Fisher Scientific; Madrid, Spain) supplemented with 10% fetal bovine serum (FBS), 100 U/mL penicillin, and 100 μg/mL streptomycin. THP-1 cells were plated at a density of 3 × 10^5^ in 24-well culture plates and differentiated to macrophages through a first incubation with 100 nM phorbol 12-myristate13-acetate (PMA) (Sigma-Aldrich, St. Louis, MO, USA) for 24 h. The PMA-containing media was discarded and replaced with fresh media without PMA for a further 24 h. 

The human epithelial cell line BICR-18 (from larynx squamous cell carcinoma) and CAPAN-2 (from pancreatic ductal adenocarcinoma) were obtained from American Type Cell Collection (ATCC, Manassas, VA, USA) and maintained at 37 °C in DMEM GlutaMAX™ (Fisher Scientific; Madrid, Spain) supplemented with 10% FBS, 100 U/mL penicillin, and 100 μg/mL streptomycin. Cultures were split every 3 days by trypsinization with 0.1% trypsin in Ca^2+^/Mg^2+^-free phosphate-buffered saline (PBS) containing 0.9 mM ethylenediaminetetraacetic acid (EDTA) (Sigma-Aldrich; St. Louis, MO, USA). 

All cells were cultured at 37 °C in humidified atmosphere of 95% air and 5% CO_2_.

### 2.2. Experimental Animals

Male Wistar rats weighing 200–250 g (7–9 weeks old) were housed in a controlled environment with free access to standard laboratory pelleted formula (A04; Panlab, Barcelona, Spain) and tap water. A period of one week was allowed for animals to acclimatize before any experimentation. All procedures were conducted in accordance with European Union regulatory standards for animal experimentation (Directive 2010/63/EU on the protection of animals used for scientific purposes). The Ethical Committee for Animal Experimentation (CEEA, Directive 396/12, University of Barcelona) approved the animal experiments (ethic approval number: 211/18, University of Barcelona, 11/04/2018).

### 2.3. Exosome Isolation

In order to generate exosome-free media, exosomes present in FBS were removed by overnight centrifugation at 100,000× *g* followed by filtration through 0.2-μm syringe-fitted filters (Millipore, Burlington, MA, USA). This exosome-depleted FBS was used for cell culture (corresponding culture media supplemented with 10% exosome-free FBS). For exosome isolation, BICR-18 cell supernatants and plasma samples from AP-induced rats were collected and centrifuged at 2000× *g* and 10,000× *g* for 10 and 30 min, respectively, at 4 °C. The last supernatant was filtered through a 0.22-µm syringe filter (Millipore, Burlington, MA, USA) and ultra-centrifuged at 120,000× *g* for 70 min. After that, the pelleted vesicles were washed with PBS and centrifuged again at 120,000× *g* [16].

The quality of exosome preparation was verified by nanoparticle tracking analysis and by determining the presence of the exosomal marker TSG101 and the absence of calnexin by Western blot. The number of exosomes obtained was also quantified by measuring their protein content using a Bradford assay (Bio-Rad Laboratories, Hercules, CA, USA).

### 2.4. Exosomes and Cell Staining 

For internalization assays, exosomes were isolated from BICR-18 cells (ExoB) or from AP-induced rats (ExoAP) and labelled with the PKH26 red fluorescent cell linker dye (Sigma-Aldrich, St. Louis, MO, USA) for 5 min. The staining reaction was stopped with 3% bovine serum albumin (BSA) for 1 min. In order to remove the unbound dye, exosomes were washed three times with PBS using 300 kDa Nanosep centrifugal devices (Pall Corporation, New York, NY, USA). Fixed cells were also stained with the DNA-specific blue, fluorescent stain 4′, 6-diamidino-2-phenylindole (DAPI) (Sigma-Aldrich, St. Louis, MO, USA) for 1–5 min at room temperature. In some experiments, THP-1 macrophages were stained with the PKH67 green, fluorescent cell linker dye for general cell membrane.

### 2.5. Cell Treatments 

To analyze exosome uptake in the presence of PEG35, differentiated THP-1 cells were incubated with 4% PEG35 diluted in PBS for 30 min prior to treatment with the appropriate stimuli: 10 µg/mL PKH26-labelled ExoB or 10 µg/mL PKH26-labelled ExoAP for 1.5 h. As well, epithelial BICR-18 and CAPAN-2 cells were incubated with 4% PEG35 for 30 min prior to treatment with PKH26-labelled ExoB for 1.5 h. The concentration of exosomes was selected according to previous in vitro studies [17]. Exosome internalization was analyzed by confocal microscopy imaging or by flow cytometry.

To analyze the modulation of the inflammatory response induced by PEG35, differentiated THP-1 cells were incubated with increasing concentrations of PEG35 (1, 2, 4, and 6%) for 30 min prior to treatment with the appropriate stimuli: 10 µg/mL ExoAP or 0.1 µg/mL lipopolysaccharide (LPS) (Sigma-Aldrich, St. Louis, MO, USA) for another 1.5 h. In another set of experiments, to determine the nuclear shift of p65 subunit of Nuclear Factor Kappa B (NFκB), differentiated THP-1 cells were incubated with 4% PEG35 for 30 min prior to treatment with 10 µg/mL ExoAP or 0.1 µg/mL LPS for another 30 min.

### 2.6. Nanoparticle Tracking Analysis

The size distribution and concentration of exosomes were measured using a NanoSight LM10 machine (NanoSight, Salisbury, UK). All parameters of the analysis were set at the same values for all samples, and three one-min-long videos were recorded in all cases. The background was measured by testing filtered PBS, which revealed no signal.

### 2.7. Animal Model of AP 

Rats (*n* = 5) were anesthetized with an intraperitoneal injection of pentobarbital at a dose of 50 mg/kg. After midline laparotomy, a polyethylene catheter connected to an infusion pump was inserted through the duodenum via the Ampulla of Vater, 3–4 mm into the biliopancreatic duct. A bulldog clamp was applied to the proximal biliopancreatic duct (near the liver) to prevent infusion into the liver. The experimental model of AP was induced in the rats by retrograde injection of 5% sodium taurocholate in saline solution at 1 mL/kg for 1 min using an infusion pump (Harvard Instruments, Edenbridge, UK). Buprenorphine (0.05 mg/kg) was intravenously administered as an analgesic immediately before surgery. Three hours after AP induction, the animals were euthanized and blood was collected in heparinized syringes from the vena cava for exosome isolation.

### 2.8. Confocal Microscopy 

Cells were imaged using an inverted Nikon Eclipse Ti2-E microscope (Nikon Instruments, Melville, NY, USA) attached to the spinning disk unit Andor Dragonfly. For all experiments, an oil-immersion objective (Plan Fluor 20×, numerical aperture (NA) 0.75, oil) was used. Samples were excited with 405 nM, 488 nM, and 561 nM laser diodes. The beam was coupled into a multimode fiber going through the Andor Borealis unit reshaping the beam from a Gaussian profile to a homogenous flat top. From there, it was passed through the 40-µm pinhole disk. Cells were imaged on a high-resolution scientific complementary metal oxide semiconductor (sCMOS) camera (Zyla 4.2, 2.0 Andor, Oxford Instruments Company, Concord, MA, USA). Fusion software from Oxford Instruments Company was used for acquisition of images. Image deconvolution was performed after acquisition. Image processing and analysis were performed with Image J/Fiji open source software using Image J Macro Language.

### 2.9. SDS-PAGE and Western Blot

For characterization of the exosomes, exosomal proteins were extracted in ice-cold radioimmunoprecipitation assay (RIPA) buffer in the presence of protease inhibitors. Extracts were then centrifuged at 15,000× *g* for 20 min at 4 °C, and the supernatants were collected. Cell lysates, used as a negative control, were obtained from differentiated THP-1 cells. Protein concentrations of the supernatants were determined by the Bradford protein assay (Bio-Rad Laboratories, Hercules, CA, USA). SDS-PAGE was performed on a 10% gel, and proteins were transferred to a polyvinylidene difluoride (PVDF) membrane for blotting (Bio Rad, Hercules, CA, USA). Membranes were blocked for 1 h in 5% non-fat milk in PBS, followed by overnight incubation at 4 °C with the following antibodies from Proteintech (Manchester, UK): anti-rabbit TSG101 (1:1000 dilution, reference 14497-1-AP) and anti-rabbit calnexin as a negative control (1:1000 dilution, reference 10427-2-AP).

To determine NFκB activation, the nuclear extract from treated THP-1 cells was prepared according to the protocol described in the nuclear extraction kit from Active Motif (Carlsbad, CA, USA). Nuclear protein concentration was determined, and SDS-PAGE was performed on a 10% gel. Then, proteins were transferred to a PVDF membrane for blotting. Membranes were blocked for 1 h in 5% non-fat milk in PBS, followed by overnight incubation at 4 °C with anti-rat p65 antibody (1:400 dilution; reference sc-372; Santa Cruz Biotechnology, Santa Cruz, CA, USA), or were blocked for 1 h in 5% BSA in PBS for subsequent overnight incubation with anti-mouse TATA binding protein (TBP) antibody (1:500 dilution; reference ab818; Abcam, Cambridge, UK).

All blots were washed and incubated with the corresponding horseradish peroxidase (HRP)-conjugated secondary antibody. Bound antibodies were detected using enhanced chemiluminescence (ECL) (Bio-Rad Laboratories, Hercules, CA, USA), and were analyzed using ChemiDoc™ Touch Imaging System (Bio-Rad Laboratories, Hercules, CA, USA).

### 2.10. Immunofluorescence

To determine NFκB or Signal transducer and activator of transcription 3 (STAT3) nuclear translocation, THP-1 macrophages were incubated in coverslips overnight at 37 °C under 5% CO_2_ in air. Treated cells were fixed with 3.5% formaldehyde for 5 min at room temperature and permeabilized with Triton X-100. The cells were stained with the following antibodies from Santa Cruz (Santa Cruz Biotechnology, Santa Cruz, CA, USA): anti-NFκB p65 (1:400 dilution, reference sc-372) and anti-STAT3 antibody (1:400 dilution, reference sc-483). Alexa Fluor 488-conjugated anti-goat antibody (Molecular Probes, Eugene, OR, USA) was used as a secondary antibody. Localization of NFκB and STAT3 was examined by confocal microscopy imaging.

### 2.11. Real-Time qRT-PCR

The total RNA from the cells was extracted with Nucleozol reagent (Macherey-Nagel, Dueren, Germany) according to the manufacturer’s protocol. RNA concentration and quality were measured with the optical density (OD) A260/A280 ratio and OD A260/A230 ratio respectively, and the integrity of the 18S and 28S ribosomal bands for all RNA preparations was verified by running a 1% agarose gel electrophoresis. Reverse transcription was conducted on a 1-µg RNA sample using the iScript cDNA Synthesis Kit (Bio-Rad Laboratories, Hercules, CA, USA). Subsequent PCR amplification was conducted using SsoAdvanced™ Universal SYBR^®^ Green Supermix (Bio-Rad Laboratories, Hercules, CA, USA) on a CFX Real-Time PCR Detection System (Bio-Rad Laboratories, Hercules, CA, USA) using 10 µL of amplification mixtures containing 50 ng of reverse-transcribed RNA and 250 nM of the corresponding forward and reverse primers. PCR primers for the detection of Interleukin 1β (IL1β) and glyceraldehyde-3-phosphate dehydrogenase (GAPDH) were designed with Primer3.0 plus [18]. The sequences were as follows: IL1β forward: 5′-GGACAAGCTGAGGAAGATGC-3′ and reverse: 5′-TCGTTATCCCATGTGTCGAA-3′, and GAPDH forward: 5′-GATCATGAGCAATGCCTCCT-3′ and reverse: 5′-TGTGGTCATGAGTCGTTCCA-3′. The specificity of the amplicons was determined by melting curve analysis. Reactions were carried out in duplicate, and threshold cycle values were normalized to GAPDH gene expression. The ratio of the relative expressions of target genes to GAPDH was calculated by the ΔCt formula.

### 2.12. Flow Cytometry

For flow cytometry analysis, differentiated THP-1 cells were pretreated with increasing concentrations of PEG35 and then co-incubated with PKH26-labeled ExoAP. Subsequently, cells were washed with PBS and trypsinized. Pelleted cells were fixated in 4% paraformaldehyde and incubated for 15 min at 4 °C. After incubation, cells were washed again with PBS and resuspended in 0.3 mL of PBS buffer for flow cytometry analysis. The PKH26 fluorescence was measured on a BD LSR Fortessa SORP cytometer using the FacsDiva software (BD Biosciences, San Jose, CA, USA). Data analyses were performed with FlowJo software (version 4.4.1; FlowJo LLC, Ashland, OR, USA).

### 2.13. IL1β Immunoassay

Interleukin-1 β (IL1β) was measured in the supernatant of treated THP-1 macrophages using a commercially available ELISA Kit (R&D Systems, Minneapolis, MN, USA) in accordance with the manufacturer’s instructions. Briefly, standards and samples reacted with a specific antibody against IL1β immobilized in a microplate. Another antibody specific for human IL1β conjugated to HRP was then added to the wells. After washing, a substrate solution was added, yielding a yellow product. The intensity of the color measured is in proportion to the amount of IL1β. The optical density was measured at 450 nM using an automated microplate reader (iEMS Reader MF; Labsystems, Helsinki, Finland). IL1β levels were obtained in pg/mL. All samples were run in duplicate.

### 2.14. Statistical Analysis 

All data were exported into Graph Pad Prism 4 (GraphPad Software, Inc.) and presented as means ± SEM. Statistical analyses were carried out by one-way analysis of variance (ANOVA), followed by Tukey’s multiple comparison test to determine the significance between pairs. The minimal level of statistical significance was <0.05.

## 3. Results

### 3.1. Biological Characterization of Isolated Exosomes

**Figure 1:**
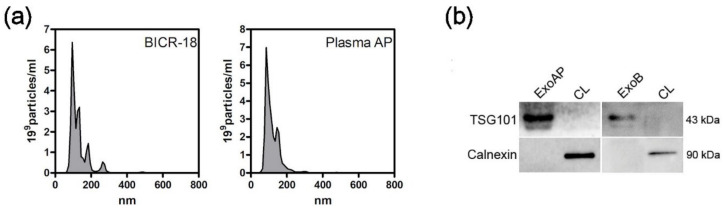
Characterization of extracellular vesicles: (**a**) Size distribution curves, evaluated by NanoSight, indicated that sizes are compatible with exosomes. (**b**) Western blot analysis was performed with whole cell lysates (CL) and pooled exosomes isolated from plasmatic acute pancreatitis-induced rats (ExoAP) and from epithelial cell line BICR-18 (ExoB) to confirm the presence of classical exosome marker (TSG101) and the absence of endoplasmic reticulum contamination (calnexin). ExoAP, exosomes from the plasma of acute pancreatitis-induced rats; CL, cell lysates; and ExoB, exosomes from BICR-18.

After collection of ExoB and ExoAP, we confirmed their size by the NanoSight particle tracking assay (Figure 1a). The size distribution of all tested vesicle preparations showed a peak between 100 and 200 nM, consistent with exosomes. Analysis by immunoblotting confirmed the presence of exosome marker TSG101 and the absence of calnexin in both exosomes from BICR-18 and from acute pancreatitis plasma origin (Figure 1b). Calnexin was readily detectable in whole cell lysates.

### 3.2. PEG35 Increased Exosome Uptake into Macrophages 

**Figure 2:**
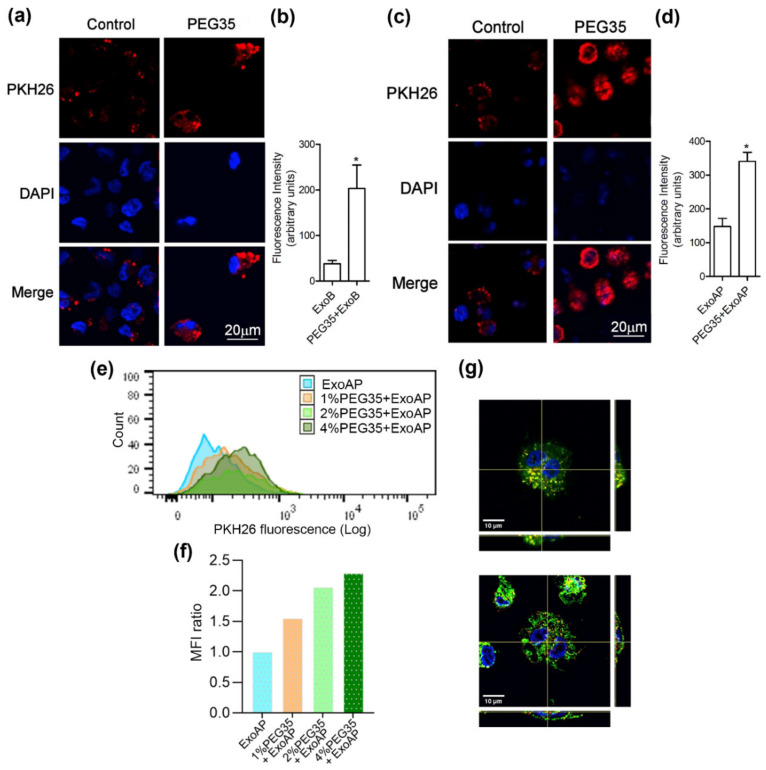
PEG35-enhanced uptake of exosomes by THP-1 macrophages: (**a**) representative confocal microscopy images showing internalized PKH26-labelled ExoB by THP-1 macrophages subjected to 4% PEG35 treatment; (**b**) fluorescence intensity analysis of the PKH26-labelled ExoB; (**c**) representative confocal microscopy images showing internalization of PKH26-labelled ExoAP by macrophages incubated with 4% PEG35; (**d**) fluorescence intensity analysis of the PKH26-labelled ExoAP, with Untreated and PEG35-treated cells fixed and imaged with confocal microscopy (scale bar, 20 µm; blue, DAPI stained nuclei; and red, PKH26 stained exosomes); (**e**) flow cytometry quantification of internalized PKH26-labeled ExoAP by macrophages at increasing concentrations of PEG35; (**f**) Median Fluorescence Intensity (MFI) normalized to the ExoAP group; and (**g**) confocal microscopy images of internalized exosomes by macrophages (z-stack projection) with orthogonal cross sections (x and y directions). Up, THP-1 macrophages incubated with exosomes; down, THP-1 macrophages pretreated with PEG35 and co-incubated with exosomes; and scale bar, 10 µm. Nuclei are reported in blue (DAPI), exosomes are in red (PKH26), and membranes are in green (PKH67). The values shown represent the mean ± SEM. * *p* < 0.05 versus ExoB or ExoAP. PEG35, 35-kDa polyethylene glycol; ExoB, exosomes from BICR-18 cells; ExoAP, exosomes from AP-induced rats; and MFI, Median Fluorescence Intensity.

To determine the ability of macrophages to internalize the isolated vesicle population, exosomes were stained with PKH26, a lipophilic fluorescent dye used regularly for exosome uptake tracking. Differentiated THP-1 macrophages were incubated with PKH26-labelled ExoB in the presence of 4% PEG35. As shown in Figure 2a,b, PEG35 enhanced the ExoB uptake capacity of macrophages. Additionally, we investigated whether PEG35 also reinforced the internalization of exosomes from acute pancreatitis. Using an experimental model of taurocholate-induced AP in rats, exosomes were collected from the bloodstream and uptake of these nanovesicles by macrophages was compared in the absence or presence of 4% PEG35. The results indicate that PEG35 also raised the macrophage capacity to internalize exosomes from AP-induced rats (Figure 2c,d). To further test PEG35’s effect on exosome internalization, THP-1 macrophages were incubated with PKH26-stained ExoAP and with increasing doses of PEG35. The amount of fluorescence captured by the cells was analyzed by flow cytometry. Quantification analysis strengthened the confocal microscopy results, demonstrating a dose-dependent effect of PEG35 treatment on the uptake of ExoAP by macrophages (Figure 2e,f). Moreover, we verified the intracellular localization of exosomes using orthogonal views reconstructed from a confocal image (Figure 2g). In this case, THP-1 macrophages were stained with the PKH67 green fluorescent dye for general cell membrane. The orthogonal projections indicated that, in both untreated and PEG35-treated cells, exosomes are distributed in the cytoplasm.

### 3.3. PEG35 Enhanced Exosome Uptake into Epithelial Cells 

**Figure 3:**
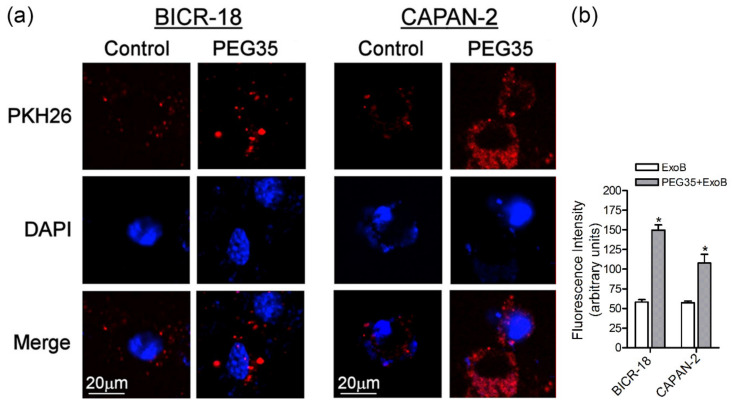
PEG35 enhanced the uptake of exosomes by epithelial cells. (**a**) Representative images showing internalized ExoB by epithelial BICR-18 and CAPAN-2 cells under treatment with 4% PEG35: untreated and PEG35-treated cells were fixed and then were imaged with confocal microscopy (scale bar, 20 µm; blue, DAPI stained nuclei; and red, PKH26 stained exosomes). (**b**) Fluorescence intensity analysis of the PKH26-labelled exosomes: the values shown represent the mean ± SEM. * *p* < 0.05 versus ExoB. Data is representative of several repeated experiments. ExoB, exosomes from BICR-18 cells; PEG35, 35-kDa polyethylene glycol.

Tissue macrophages are known to express a wide range of surface molecules for recognition and uptake of host-derived and foreign particles by phagocytosis and for clearance of soluble molecules by endocytosis [19]. Moreover, exosomes are known to internalize more efficiently by phagocytic cells than non-phagocytic cells [20]. Hence, we also evaluated the effect of PEG35 in exosome uptake by epithelial BICR-18 and CAPAN-2 cells. As occurs with macrophages, the presence of 4% PEG35 significantly increased ExoB cell internalization by both epithelial cells (Figure 3a,b).

### 3.4. PEG35 Modulated the Inflammatory Response Induced by AP-Derived Exosomes in Macrophages

**Figure 4:**
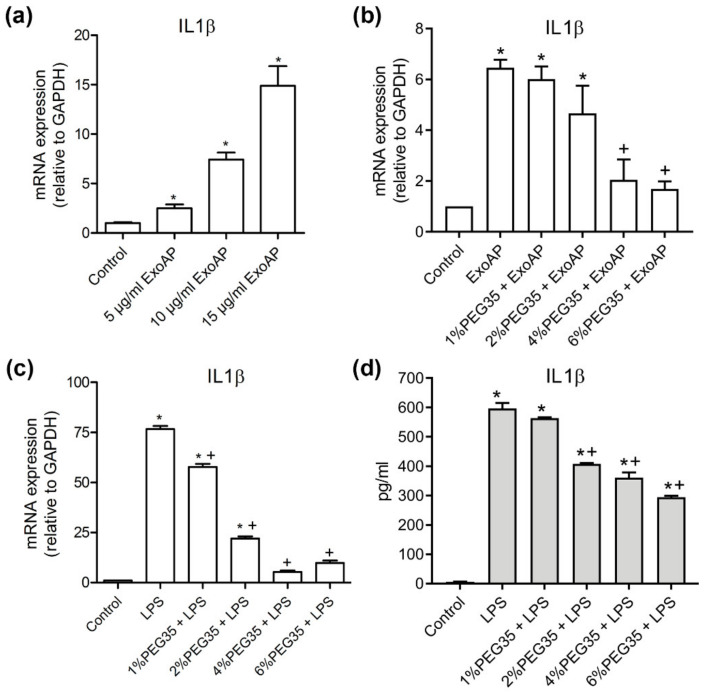
Expression of pro-inflammatory Interleukin 1β (IL1β) in THP-1-treated cells: (**a**) Gene expression by real-time qRT-PCR of IL1β in THP-1 macrophages treated with increasing concentrations of ExoAP; (**b**) gene expression by real-time qRT-PCR of IL1β by THP-1 cells subjected to increasing concentrations of PEG35 and co-incubated with ExoAP; (**c**) IL1β gene expression by real-time qRT-PCR in THP-1 macrophages subjected to increasing concentrations of PEG35 and co-incubated with lipopolysaccharide (LPS), where, in all cases, mRNA induction levels were normalized to glyceraldehyde-3-phosphate dehydrogenase (GAPDH) mRNA expression; and (**d**) expression levels of IL1β protein released by THP-1 cells pretreated with increasing concentrations of PEG35 and then co-incubated with LPS. Bars represent the mean values of each group ± SEM. * *p* < 0.05 versus control, + *p* < 0.05 versus ExoAP or LPS. Each determination was carried out in triplicate. ExoAP, exosomes from acute-pancreatitis-induced rats; LPS, lipopolysaccharide; PEG35, 35-kDa polyethylene glycol; and IL1β, interleukin 1β.

The previous results of our group support an anti-inflammatory role for PEG35 against AP-associated inflammation [15] and that exosomes play a pro-inflammatory role in experimental AP [14]. To investigate the potential role of this polymer in the modulation of the inflammatory response induced by AP-derived exosomes, we first confirmed in vitro the pro-inflammatory role of circulating exosomes from AP on THP-1 macrophages (Figure 4a). Macrophages treated with increasing concentrations of ExoAP triggered an evident dose-dependent inflammatory response through the increase of IL1β. Furthermore, we evaluated the effect of PEG35 on this activation. The induction of IL1β gene expression activated by an intermediate dose of 10 μg/mL of ExoAP was inhibited by the presence of PEG35 in a dose-dependent manner (Figure 4b). The same inhibitory effect was observed when PEG35 was administered to macrophages directly activated with LPS (Figure 4c), pointing to a nonspecific anti-inflammatory role of PEG35. Finally, we corroborated that the upregulated protein levels of IL1β by LPS-treated macrophages are inhibited by the presence of PEG35 dose-dependently, thus confirming that this polymer modulates IL1β expression at both the mRNA and protein levels (Figure 4d). 

### 3.5. PEG35 Reduced Pro-Inflammatory Response in Macrophages through the Inhibition of NFκB 

One of the important features of the anti-inflammatory effect of PEG35 is that it counteracts the activation of genes encoding inflammatory mediators, such as TNFα, IL1β, or IL6. Because NFκB is highly active both in inflammatory cells, such as macrophages, and in cells found in inflamed tissues, it is recognized as a key mediator of inflammation. In addition to NFκB, STAT3 signaling also plays a pivotal role in inflammatory processes. Both factors are essential signaling molecules that coordinate inflammatory response in front of different stimuli.

**Figure 5:**
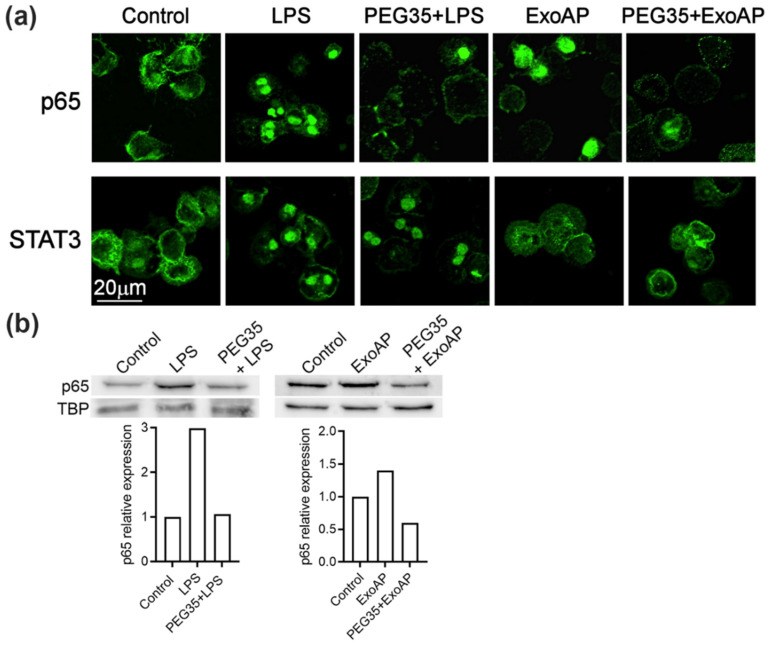
PEG35 suppressed p65 translocation to the nucleus in LPS or ExoAP-stimulated macrophages: (**a**) representative images of immunofluorescence staining for subcellular localization of the p65 subunit of Nuclear Factor Kappa B (NFκB) and Signal transducer and activator of transcription 3 (STAT3) observed by confocal microscopy, with scale bar 20 µm, and (**b**) nuclear expression of p65 assessed by Western blot analysis. TBP expression was used as the loading control. Densitometry quantification of the Western blot was performed for p65. Data are representative of several repeated experiments. LPS, lipopolysaccharide; ExoAP, exosomes from AP-induced rats; PEG35, 35-kDa polyethylene glycol; and TBP, TATA binding protein.

Given that the molecular mechanisms underlying the anti-inflammatory role of PEG35 need to be elucidated, we examined the effect of PEG35 on these pathways in macrophages. As shown in Figure 5a, unstimulated THP-1 macrophages showed that the p65 subunit of NFκB staining localized in the cytosol. After LPS or ExoAP stimulation, p65 staining was mostly transferred to the nucleus, indicating the activation of this signaling pathway. Pretreatment of cells with 4% PEG35 inhibited p65 accumulation in the nucleus. On the other hand, STAT3 was activated by LPS treatment but not by ExoAP and the activation induced by LPS was not inhibited by PEG35. The inhibitory effect of PEG35 on NFκB was confirmed by measuring the nuclear levels of p65 subunit by Western blot (Figure 5b). The small increase found in the ExoAP group was expected considering that a similar small rise was found in IL1β mRNA synthesis in contrast to the higher levels of IL1β upon LPS stimulation.

## 4. Discussion

Over the last decade, considerable interest has grown in the study of PEG properties and their use has extended to different fields. Recently, our group has demonstrated the anti-inflammatory effect of prophylactic administration of PEG35 in an experimental model of acute necrotizing pancreatitis, and besides, we have shown that therapeutic treatment with this polymer exerted significant protection against acute lung inflammation associated with this disease [15].

In the current study, we explored whether this protective action of PEG35 is related to effects on the cellular uptake of exosomes. It is well known that these nanovesicles are able to trigger the inflammatory response in a broad spectrum of pathologies. In relation to the pancreas, there is scarce bibliographical documentation on the role of exosomes in pancreatitis-associated inflammation. Our group has previously reported that circulating exosomes may act as pro-inflammatory mediators and may activate alveolar macrophages in experimental AP [14]. More recently, another mechanism of exosome-induced inflammation in AP have been elucidated in which NLRP3 inflammasome activation and subsequent pyroptosis in alveolar macrophages are responsible for the lung injury secondary to AP [21].

Since PEG has the capability to bind biological membranes [22], it could be hypothesized that its presence impairs the process of exosome uptake, thus reducing the associated inflammatory response triggered by these nanovesicles. Unexpectedly, our results indicate the opposite mechanism and exosome uptake increased in a dose-dependent manner in the presence of PEG35.

We then explored whether this mechanism was related to the high phagocytic capacity of macrophages and evaluated the effect of PEG35 on different epithelial cell lines. In all cases, the presence of 4% PEG35 increased exosome uptake, pointing to a nonspecific mechanism which probably involves the binding process between exosomal and cell membranes.

Our group first clarified that exosomes are involved in AP and that circulating exosomes transform alveolar macrophages into a pro-inflammatory phenotype. This change seems to be related to the content of exosomes, which has been found to be enriched in immune response-associated proteins such as S100-A8, S100-A9, Ficolin-2, or LPS-binding protein [14]. In addition, exosomes released from taurolithocholate-treated pancreatic acinar cells contain differentially expressed miRNAs, which promote inflammation in macrophages through NF𝜅B activation [23]. Therefore, it seems clear that the exosomes from AP carry compounds with relevant pro-inflammatory activity that significantly contributes to disease progression.

As expected, our results confirm that exosomes from AP increased the expression of pro-inflammatory IL1β on THP-1 macrophages, revealing their activation. Interestingly, PEG35 treatment succeeded in the inhibition of these inflammatory cytokine’s expressions. This fact suggests that, despite the increase in internalization of exosomes induced by PEG35, its anti-inflammatory effect is able to prevent the activation induced by these nanovesicles.

Next, we determined the direct anti-inflammatory effect of PEG35 on the activation of macrophages and confirmed that this polymer markedly prevented IL1β mRNA and protein expression induced by LPS treatment in a concentration-dependent manner. This is consistent with the results obtained in a previous study in which we demonstrated a direct anti-inflammatory effect of PEG35 in pancreatic acinar AR42J cells [24]. In this sense, we found that administration of this polymer attenuated the expression of pro-inflammatory cytokines and associated cell death markers following TNFα or cerulein treatment.

Given that activation of NFκB and STAT3 plays a crucial role in inflammatory processes, it was conceivable that these signaling pathways could be involved in PEG35 inhibition of inflammation. Although triggered by different pathways, activated STATs and NFκB translocate into the nucleus and function either individually or collaboratively in regulating inflammatory gene expression. Our data indicated that macrophage treatment with PEG35 caused a shift in the p65 nuclear localization observed under LPS or ExoAP stimulation. However, STAT3 was not found to be a target for PEG35-mediated attenuation of inflammation in LPS-treated macrophages. The direct inhibitory effect of PEG35 on NFκB allows for understanding the fact that, although more exosomes are uptaken, thus implying a greater pro-inflammatory stimulus, PEG35 inhibits its activation at a subsequent stage and the outcome is a lower expression of inflammatory mediators.

## 5. Conclusions

PEG35 facilitates exosome internalization into cells but can prevent the pro-inflammatory effect of AP-derived exosomes by targeting the NFκB signaling pathway. Nevertheless, our results indicate that the effects of PEG35 related to exosomes must be carefully considered. The increase in exosome uptake could be attractive in situations where exosomes carry molecules of interest, as occurs in the anti-inflammatory effects of exosomes released by mesenchymal stem cells [25]. However, in other pathological situations in which exosomes contribute to the pathogenic process, any increase in exosome uptake could be detrimental. In addition, the direct anti-inflammatory effect exerted by PEG35 blocking activation of NFκB must be taken into account. Obviously, further studies are required in order to clarify both the potential and limitations of PEGs when using them in pathologies in which exosomes play a relevant role.
